# Optimization and Application of A Bionic System of Dynamic Co-Culture with Hepatocytes and Renal Cells Based on Microfluidic Chip Technique in Evaluating Materials of Health Food

**DOI:** 10.3390/nu14224728

**Published:** 2022-11-09

**Authors:** Di Chen, Jiyong Yin, Junsheng Huo, Jing Sun, Jian Huang, Tiantong Li, Chaoqun Sun, Zhuo Yang, Wen Qin

**Affiliations:** National Institute for Nutrition and Health, Chinese Center for Disease Control and Prevention, Beijing 100050, China

**Keywords:** bionic system, co-culture of hepatocytes and renal cells, microfluidic chip, emodin, raw material of health food

## Abstract

We aimed to explore the optimization and application of a bionic system of dynamic co-culture with hepatocytes and renal cells based on the microfluidic chip technique in evaluating emodin, which might replace the conventionally cytological evaluation technique of health food. After optimal experiments, the improved bionic system was composed of human hepatocellular carcinoma cells (HepG2), human renal glomerular endothelial cells (HRGECs), rat tail collagen type I, and gelatin with optimized concentrations (1.3 mg/mL + 7.5%). The applicability of the bionic system indicated that the growth stability was appropriate (CV: 7.36%), and the cell viability of that gradually decreased with the increasing of emodin concentration from 0–100 μM, which statistic significances were at 50 and 100 μM (*p* < 0.05), and the stained results of dead/live cells also showed the same trend. The LDH level appeared rising trend after decline between 0 μM and 100 μM emodi, and the level of that at 100 μM emodin was significantly higher than that at 25 μM and 50 μM emodin, respectively. The BUN level continuously and significantly declined with the increasing of emodin concentration (*p* < 0.05). Our research realized the application of this optimized bionic system in evaluating emodin, and provided a useful platform and reference for further in vitro alternative research with regard to evaluating the efficacies of health food in the future.

## 1. Introduction

The evaluation of health food is an important field that is improving gradually, while the conventional evaluation technique that might reduce the reliability of the function of health food has been not able to meet the requirements of evaluating health food. The conventional evaluation techniques of health food mainly include human trials, animal experiments and in vitro static cytological experiments in a culture dish or plate [1]. They once provided important support in evaluating health food, while their disadvantages also are obvious. The difficulties of human trials are mainly their high cost and limited sample resources, and the controversial elements of animal experimentation include species differences between animals and humans, they are time-consuming, and some interference factors are uncontrollable [2,3]. The conventionally cytological experiment includes static mono-culture of a single kind of cell line and a static co-culture of multiple kinds of cell lines. The disadvantages of that include five aspects: a single kind of cell line is not able to completely simulate the real status of the human body [4]; the interaction between cells and the matrix or among different kinds of cell lines are often covered, [5]; the cell plate of static cell culture mode often leads to the deformation of the original structures of cells [3]; the static culture condition cannot simulate the gradient concentration-induced reaction process in cell microenvironment [3]; and the manual operation might reduce the stability and authenticity of experimental results [3]. In view of the above problems, it is urgent to optimize and apply a new kind of improved bionic system of dynamic co-culture with multiple types of cells so as to more accurately explore the true effects of raw materials of health food in the human body.

The microfluidic chip technique can provide a new kind of cytological research platform of dynamically bionic culture. It breaks through the limitations of conventional cytological experiments and can accurately conduct dynamic culture by the perfusion technique, which is operated by controlling pressure and temperature in the micro channel of a microfluidic chip [6,7]. 

Our research team once successfully constructed a hepatocyte model that evaluated the efficacies of health food by using the microfluidic chip technique at an earlier stage of this research, which attempted to avoid the disadvantages of a conventional petri dish or plate mode [8]. The constructed hepatocyte model indicated a potential that the microfluidic chip technique could provide an in vitro alternative experiment in evaluating the efficacies of health food, while it still could not entirely simulate the metabolic process of the ingredients of health food in the human body because it only contained hepatocytes. We also once planned to conduct co-cultured experiments with hepatocytes and renal cells under conventionally static culture conditions by using the Millicell cell culture inserts technique; however, this hypothesis finally had to be given up because the static co-culture mode could not thoroughly reach the bionic effect of dynamic co-culture.

In this research, our team attempted to improve and optimize the operation condition of a bionic system of dynamic co-culture with HepG2 cells and human renal glomerular endothelial cells (HRGECs) based on the microfluidic chip technique and then to apply this bionic system to evaluate emodin as a representative material of health food.

Emodin (1,3,8-trihydroxy-6-methylanthraquinone) is a derivative of hydroxyanthraquinone, which has a relative molecular weight of 270.23 g/mol [8]. It is one of the effective components of *Rheum Palmatum* L., *Rheum tanguticum Maxim.ex Regel* or *Rheum officinale Baill*. of the *Polygonaceae* herbaceous plant [9], and there are approximately 800 kinds of traditional Chinese medicinal materials containing emodin according to the introduction of the *Pharmacopoeia of the People’s Republic of China*. In health food, emodin is a typical material which can affect both the liver and kidney in its metabolic process. Lv [10] once reported that emodin from the *Fallopia multiflora* (*Thunb.*) *Harald* could have an apoptosis-induced effect on the human hepatocyte cell line L-02, and the effect of emodin depended on concentration and time of exposure, and it could damage the subcellular structure of the hepatocytes of rats. Zhang [11] once pointed out that emodin could accelerate the apoptosis of HepG2 cells via vacuolization and the reduced membrane potential of mitochondria. For kidney, Yan M. [12] once provided evidence that the concentrations of β-2 microglobulin and urinary albumin would sharply increase after 13 weeks with emodin exposure, and the results of Wang [13,14] indicated that emodin could lead to a G1 block in a human proximal renal tubular epithelial cell line (HK-2) and could cause apoptosis through the caspase-3 dependent pathway. In addition, National Toxicology Program [15] accounts for a series of pathological and morphological changes in both rats and mice, including the hypertrophy of liver tissue and the generation of transparent droplets within kidney tubules after exposure with different dosages of emodin ranging from 40 to 800 mg/kg. On the other hand, some studies [9,13,16,17] showed that emodin possessed bidirectional effects on hepatocytes and renal cells, which meant emodin would protect hepatocytes and renal cells at appropriate concentrations or time, while it would damage them at high concentrations or over long times. Depending on the above analyses, emodin is chosen as an representative material in verifying whether the optimized bionic system of dynamic co-culture with HepG2 cells and HRGECs can be applied to evaluate the raw materials of health food.

Therefore, our hypotheses were whether we could improve and optimize a bionic system of dynamic co-culture with HepG2 cells and HRGECs based on the existing microfluidic chip technique, and whether this bionic system could be applied to evaluate emodin. We want to realize a bionic co-culture that can simulate the metabolic process of emodin in the human body at the greatest extent through this research. The optimized bionic system would provide a useful platform and the results would provide an important reference for in vitro alternative research that evaluates the toxicity of other raw materials of health food. As with the above hypotheses, this research conducted an evaluation of emodin exposure after the bionic system of dynamic co-culture with HepG2 cells and HRGECs based on the microfluidic chip technique that was improved and optimized.

## 2. Materials and Methods

### 2.1. Materials and Reagents

Endothelial Cell Medium (ECM) was purchased from ScienCell (San Diego, CA, USA), and fetal bovine serum (FBS) and Dulbecco’s Modified Eagle’s Medium (DMEM) (contained 1% penicillin/streptomycin solution, 1% endothelial cell growth supplement and 5% FBS) were purchased from Gibco (Grand Island, NY, USA). Trypsin-EDTA and rat tail collagen type I were purchased from Solarbio Company (Beijing, China), and gelatin was purchased from Aladdin (Shanghai, China). The live/dead kit was purchased from KeyGEN BioTECH (Jiangsu, China). The cell counting kit-8 (CCK-8) was purchased from Beyotime (Shanghai, China). Hoechst33342 (art. H1399) and CellTracker Green CMFDA (art. C7025) were purchased from Thermo Fisher Scientific (Waltham, MA, USA). Lactate dehydrogenase (LDH), alanine aminotransferase (ALT), aspartate aminotransferase (AST) and the blood urea nitrogen (BUN) assay kit were purchased from Nanjing Jiancheng (Nanjing, China). Emodin was purchased from TCI (Tokyo, Japan). All of these chemical reagents used in this experiment were of analytical reagent grade.

### 2.2. Main Instruments and Equipment

The microfluidic cell chip culture analysis platform CellASIC ONIX2 was purchased from Merck & Co., Inc. (Darmstadt, Germany); the EVOS M7000 imaging system was purchased from Invitrogen Life Technology Co., Ltd. (Carlsbad, CA, USA); the research-grade fluorescence inverted microscope ECLIPSE TS100 was purchased from Nikon Corporation (Nikon, Japan); the Allegra x-22R Centrifuge was purchased from Beckman coulter, Inc. (Brea, CA, USA); the MCO-18AIC CO_2_ Incubator was purchased from Panasonic Corporation (Tokyo, Japan); and the SpectraMax I3X Enzyme marker was purchased from Molecular Devices Instruments Ltd. (San Jose, CA, USA).

### 2.3. Cell, Culture and Proliferation

In this experiment, HepG2 cells were purchased from the Shanghai Cell Bank of the Chinese Academy of Sciences (Shanghai, China), and HRGECs were purchased from BNCC (Beijing, China). HepG2 cells and HRGECs were cultured in a DMEM and ECM cell medium at 37 °C and CO_2_ of 5% to reach cell proliferation before the optimal experiment and the application experiment of bionic system of dynamic co-culture with HepG2 cells and HRGECs was performed. The two kinds of cell lines were successively added in the cell culture chamber of microfluidic chips after the cell density of each kind of cell line reached 80–90% of the cell culture flask.

### 2.4. The Working Principle and Related Characterization of the Microfluidic Chip Instrument

**Figure 1:**
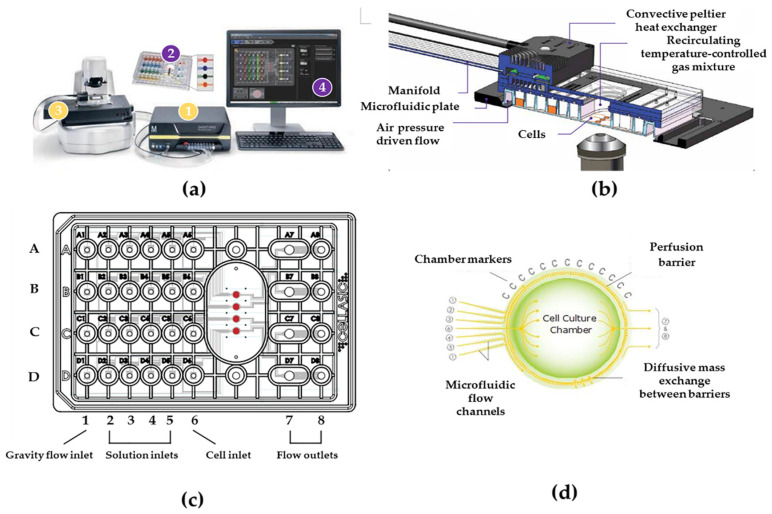
Cellasic ONIX2 microfluidic device and the structure flow diagram of Cellasic M04S culture plate: (**a**) The components of microfluidic cell chip laboratory include ① controller ② chip culture plate ③ manifold ④ control software; (**b**) Microfluidic structure and flow diagram of manifold; (**c**) The working design of Cellasic M04S culture plate; (**d**) The structure diagram of cell culture chamber.

The cellasic M04S cell culture plate is connected with the CellASIC ONIX2 microfluidic device as a microfluidic cell chip laboratory (Figure 1a) to conduct this experiment. The controller is connected to the microfluidic plate via manifold, and the gas with set temperature could be inputted directly in each cell culture chamber (Figure 1b), and each well of Cellasic M04S cell culture plate (Figure 1c) according to the designed experimental plan. In the cell culture plate, the sixth column (A6–D6) is used to add cells, the first column (A1–D1) is used to perfuse the cell culture medium, the four columns (A2–D2, A3–D3, A4–D4, A5–D5) are used to perfuse different intervention materials (such as emodin), and the two columns (A7–D7, A8–D8) are used to collect the metabolites of cells. The cell culture chambers of this plate are used to culture cells after the cells are added to them from the sixth column (A6–D6). The structure and the technique process of the cell culture chamber are shown in Figure 1d. The basic operations of liquid flow velocity, temperature and CO_2_ concentration [8], as well as the sterilization, cleaning and liquid balance of cell culture plate [18,19], are conducted according to the referred data [8] of our team at an early stage.

### 2.5. Improvement of Routine Mode of Adding Cells in Microfluidic Chip

The routine operation of the CellASIC ONIX2 microfluidic cell chip laboratory [18,19] cannot form separate and respective distributions of two kinds of cell lines in one cell culture chamber. In this research, we improved the routine operation of adding cells in a CellASIC ONIX2 microfluidic cell chip laboratory and fully utilized the advantage of reverse pressuring of the manifold on the seventh column to realize the two kinds of cells that were added respectively and successively in the same one cell culture chamber. On the first day of the experiment, the HRGECs were added into each well (2.54 × 10^6^ cells/mL, 200 μL) of the seventh column after they were digested with 0.25% trypsin in conventional culture flasks and were collected by centrifugation at 1000 r/min for 3 min. At the same time, 300 μL ECM was added into each well of the 1st column to provide the culture medium. The HRGECs were cultured successively in a cell culture chamber (4–6 h, 37 °C and 5% CO_2_) after they were added into each cell culture chamber by reverse pressure. Next, the HepG2 cells were added into each cell culture chamber from each well (2.54 × 10^6^ cells/mL, 50 μL) of the sixth column after HRGECs successfully realized the adherent culture. After that, HepG2 cells were cultured with HRGECs in the same one cell culture chamber at 37 °C and 5% CO_2_. The ECM of the first column could realize continuous and dynamic perfusion for the bionic system of dynamic co-culture with HepG2 cells and HRGECs through its gravity flow.

### 2.6. Optimization of Dilution Conditions of Different Kinds of Cell Lines in the Bionic System

After some pre-experiments of the concentration of glue were explored at the early stage of this research, the HRGECs (20 × 10^6^ cells/mL) were diluted to 54 × 10^6^ cells/mL by rat tail collagen I with differently final concentrations (1.5 mg/mL, 3.0 mg/mL and 4.0 mg/mL) + 0.1 M NaOH + 10 × PBS, and by gelatin with differently final concentrations (7.5%, 8.5% and 10%), and by mixed glue (1.3 mg/mL rat tail collagen I + 0.1 M NaOH + 10 × PBS, and 7.5% gelatin), respectively (Table 1). They were then added into each well of the seventh column before they were pressured into each cell culture chamber under the operation conditions (two times with 3 s at 0.3 psi after four times with 3 s at 0.15 psi). The optimal dilution condition of HRGECs was determined after the above dilution results were compared. The appearance of the optimal dilution effect was that HRGECs could evenly cover the lower reaches of the cell culture chamber (right of the cell culture chamber), which formed a separate part in the cell culture chamber.

The HepG2 cells (20 × 10^6^ cells/mL) were diluted to 2.54 × 10^6^ cells/mL by gelatin with differently final concentrations (2.5%, 4.0%, 5.0%, 7.5%, 8.5% and 10%) and by mixed glue (1.3 mg/mL rat tail collagen I + 0.1 M NaOH + 10 × PBS, and 7.5% gelatin), respectively (Table 1). Next, they were added into each well of the sixth column before they were pressured into each cell culture chamber under the operation conditions (four times of 2 s at 0.26 psi, two times successively with 3 s at 0.26 psi). The optimal dilution condition of HepG2 cells was determined after the above dilution results were compared. The appearance of the optimal dilution effect was that HepG2 cells could evenly cover the upper reaches of the cell culture chamber (left of the cell culture chamber), which formed another separate part in the cell culture chamber.

### 2.7. Analysis of the Distribution of HepG2 Cells and HRGECs in the Same One Cell Culture Chamber by the Cell Tracing Technique

This research adopted the cell tracing technique to further verify the distributions of HepG2 cells and HRGECs in a bionic system. For staining HRGECs, the HRGECs were incubated in an incubator (37 °C, 5% CO_2_, 50 min) after they were re-suspended to 20 × 10^6^/mL by 0.2 mL CellTracker Green CMFDA (art. C7025) (5 μΜ). After 50 min, the successfully stained HRGECs were centrifuged and re-suspended in 0.2 mL ECM. They were then further diluted to 2.54 × 10^6^ cells/mL as the optimal dilution condition (1.3 mg/mL rat tail collagen I + 0.1 M NaOH + 10 × PBS, and 7.5% gelatin) based on Section 2.6. After 4–6 h, the HepG2 cells were also incubated in an incubator (37 °C, 5% CO_2_, 10 min) after they were re-suspended to 20 × 10^6^/mL by 0.1 mL Hoechst33342 (art. H1399) (5 μg/mL). The successfully stained HepG2 cells were centrifuged and re-suspended in 0.1 mL ECM. After that, these stained HepG2 cells were also diluted to 2.54 × 10^6^ cells/mL as the optimal dilution condition (1.3 mg/mL rat tail collagen I + 0.1 M NaOH + 10 × PBS, and 7.5% gelatin).

After HRGECs and HepG2 cells were successfully stained, they were respectively and successively added into the same one cell culture chamber according to the operation of Section 2.5.

The images of the distribution of HepG2 cells and HRGECs in the same one cell culture chamber of the microfluidic chip were then imaged on an EVOS M7000 imaging system by using 350/461 (excitation/emission) and 492/517 (excitation/emission), and the corresponding cell count and area measurement was conducted by EVOS Analysis software.

### 2.8. The Growth Observation and CCK-8 Detection of the Bionic System

The observation of the growth of co-cultured HepG2 cells and HRGECs were conducted by an EVOS M7000 imaging system at the 0 h and the 6th h (the first day) after HRGECs were added into lower reaches of the cell culture chamber, and at the 0 h (the first day) and the 72nd h (the third day) after HepG2 cells were added into the upper reaches of the cell culture chamber. The cell count results of the 0 h (the first day) and the 72nd h (the third day) were recorded. Next, the CCK-8 detection was conducted at the 72nd h, and the OD value of CCK-8 detection was used to further determine the survival status of the bionic system. The mixed acetoxymethyl/propidium iodide (AM/PI) reagent (100 μL in which final concentrations of AM and PI were 2 μM and 8 μM, respectively) was then dynamically and continuously perfused into each cell culture chamber via each well of the second column within 40 min under 3.3 psi pressure. The double-stained cells were observed and photographed under 495/520 (excitation/emission) and 530/620 (excitation/emission) by an EVOS M7000 imaging system. The live/dead cells would appear green and red, respectively.

### 2.9. Influence of Emodin on Cell Viability of the Bionic System

The emodin (300 μL) with different concentrations (0, 25, 50, 100 μM) were dynamically and continuously perfused into each cell culture chamber via each well of the third column of microfluidic chips under 1.3 psi after HepG2 cells and HRGECs were co-cultured within the same one cell culture chamber for 24 h. The experiment of emodin exposure was conducted within 48 h. The metabolic products of co-cultured HepG2 cells and HRGECs under emodin exposure with different concentration were collected from each well of the 7th column of microfluidic chip to implement subsequent detection within the next 48 h. After 20 min wash with PBS, CCK-8 working solution (120 μL which was composed of CCK-8 original solution and DMEM medium without serum as 1˸10) was dynamically and continuously perfused into each cell culture chamber via each well of the fourth column under 3.8 psi pressure within 3 h, which was conducted to evaluate the influence of emodin on cell viability of the bionic system. The reaction products of each cell culture chamber were collected from each well of the seventh column after 3 h, and they were detected at 450 nm by a SpectraMax I3X Enzyme marker. Finally, the cell viability of the bionic system under emodin exposure was calculated according Formula (1), respectively.

Cell viability (%) = [A (dosed) − A (blank)] × 100/[A (0 dosed) − A (blank)](1)

A (dosing): OD value of the well with cells, CCK-8 solution and drug solution

A (0 dosing): OD value of the well with cells, CCK-8 solution but no drug solution

A (blank): OD value of the hole without cells

The evaluation of live/dead co-cultured cells was then conducted after the CCK-8 experiment. The mixed AM/PI reagent (100 μL in which final concentrations of AM and PI were 2 μM and 8 μM, respectively) was dynamically and continuously perfused into each cell culture chamber via each well of the second column of the microfluidic chip within 40 min under 3.3 psi pressure. After 40 min, the live (green)/dead(red) cells were observed and photographed under 495/520 (excitation/emission) and 530/620 (excitation/emission) by an EVOS M7000 imaging system, respectively.

### 2.10. Influences of Emodin on the Basic Functions of the Bionic System

The basic experiment operation referred to Section 2.9. The metabolites of the bionic system under emodin exposures with different concentrations were collected from each well of the seventh column after the experiment of emodin exposure was conducted within the subsequent 48 h. Next, these metabolites were used to detect the ALT, AST, LDH and BUN of this bionic system, which are able to reflect the influences of emodin on the basic functions of bionic system. The specific detection methods of these indicators should refer to the instructions of kits.

### 2.11. Statistical Analysis

All experiments were repeated according to the requirement of research design, and all data of these experiments should meet normality that was analyzed by using a Shapiro-Wilk test and equal variances. The analyzed results of these detected data were expressed as means ± standard deviation (M ± SD). A Student’s *t*-test was used to analyze the survival statuses of the bionic system, and a one-way analysis of variance (ANOVA) followed by a least significant difference (LSD) test was conducted to analyze the influences of emodin on cell viability and functions of a bionic system for multiple comparisons. A two-sided *p* value < 0.05 was considered to be statistically significant. Evos Analysis software (Invitrogen Life Technology Co., Ltd., Carlsbad, CA, USA) was adopted to perform cell count and area measurement of HepG2 cells and HRGECs in the same one cell culture chamber. Microsoft Excel 2010 (Mircosoft Inc., Redmond, Washington, DC, USA) and SPSS19.0 (IBM, Armonk, NY, USA) were implemented in the statistical analysis for calculating data and statistical graphs, which were supported by the Windows 7.0 operating system (Microsoft Inc., Redmond, Washington, DC, USA).

## 3. Results

### 3.1. Optimization of Dilution Conditions of Different Kinds of Cell Lines in Bionic System

**Figure 2:**
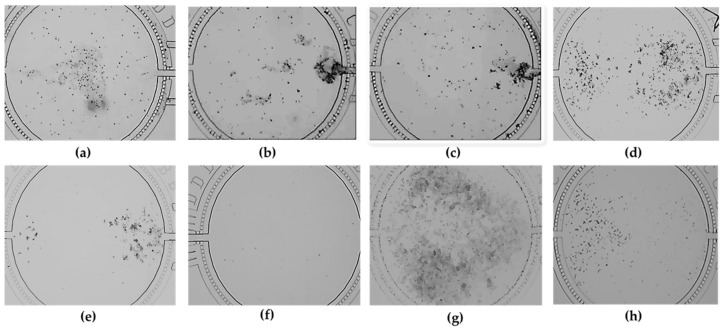
Distribution effect of the two kinds of cell lines under different dilution conditions in cell culture chamber. (**a**–**h**) subfigure corresponded to the (**a**–**h**) condition in Table 1, respectively.

The distribution results of the bionic system of dynamic co-culture with HepG2 cells and HRGECs displayed that the optimal dilution condition was mixed glue (1.3 mg/mL rat tail collagen I + 0.1 M NaOH + 10 × PBS, and 7.5% gelatin) for HRGECs, and mixed glue (1.3 mg/mL rat tail collagen I + 0.1 M NaOH + 10 × PBS, and 7.5% gelatin) for HepG2 cells (Figure 2h). The HepG2 cells and HRGECs could evenly cover the upper and lower reaches of the cell culture chamber under the above optimal dilution conditions, respectively, and the growth region of each kind of cell line was a separate part in the cell culture chamber.

The observed results also displayed single 3.0 mg/mL rat tail collagen I + 0.1 M NaOH + 10 × PBS (Figure 2b, lower reaches of the cell culture chamber), single 4.0 mg/mL rat tail collagen I + 0.1 M NaOH + 10 × PBS (Figure 2c, lower reaches of the cell culture chamber), single 8.5% gelatin, (Figure 2e) and single 10% gelatin (Figure 2f) directly blocked the microchannel of the microfluidic chip because their concentrations were too high. In addition, the two kinds of cell lines overspread the whole cell culture chamber and they would not be distinguished under single 0.0 mg/mL rat tail collagen I + 0.1 M NaOH + 10 × PBS (Figure 2g), single 1.5 mg/mL rat tail collagen I + 0.1 M NaOH + 10 × PBS (Figure 2a, lower reaches of the cell culture chamber), single 0.0% gelatin (Figure 2g), single 2.5% gelatin (Figure 2a, upper reaches of the cell culture chamber), single 4.0% gelatin (Figure 2b, upper reaches of the cell culture chamber) and single 5.0% gelatin (Figure 2c, upper reaches of the cell culture chamber), respectively.

On the other hand, the covered region of each kind of cell line also was able to be a separate part in the cell culture chamber under single 7.5% gelatin (Figure 2d), but it was not the optimal condition because the single 7.5% gelatin could not fully simulate the true microenvironment of human body and it could not entirely achieve the bionic effect of cells. Therefore, the optimal dilution condition was determined as the mixed glue (1.3 mg/mL rat tail collagen I + 0.1 M NaOH + 10 × PBS, and 7.5% gelatin).

### 3.2. Analysis of the Distribution of HepG2 Cells and HRGECs in the Same One Cell Culture Chamber

**Figure 3:**
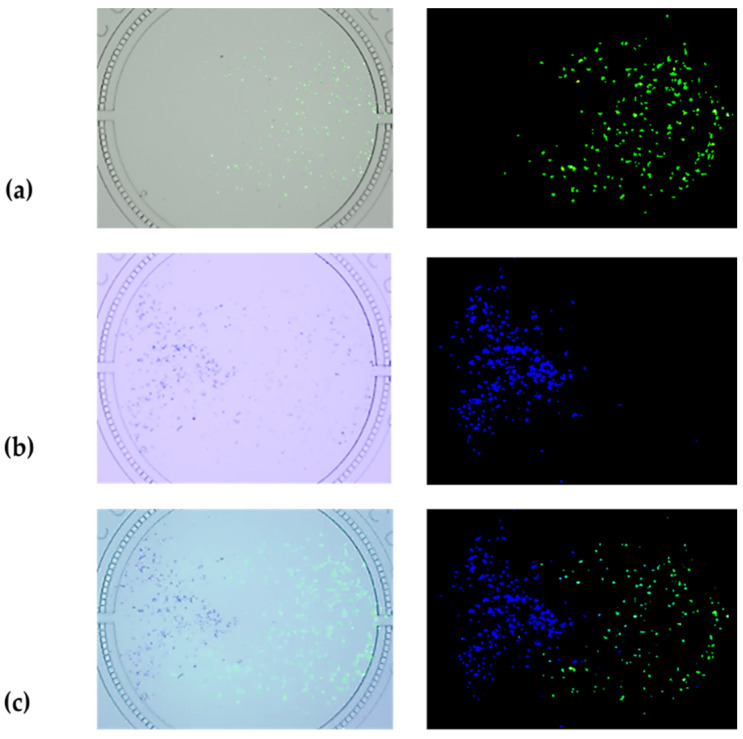
The distribution of co-cultured HepG2 cells and HRGECs in the same one cell culture chamber of microfluidic chip. (**a**) Distribution of stained HRGECs with green in cell culture chamber (The merged image of GFP channel and Trans channel, and the image of GFP channel) (×4); (**b**) Distribution of stained HepG2 cells with blue in cell culture chamber (The merged image of DAPI channel and Trans channel, and the image of DAPI channel) (×4); (**c**) Distribution of stained HepG2 cells and stained HRGECs in the same one cell culture chamber (The merged image of DAPI, GFP and Trans channels, and the merged image of DAPI channel and GFP channel) (×4).

The observed results of stained HepG2 cells (blue) and HRGECs (green) indicated that HepG2 cells and HRGECs had successfully covered the upper and lower reaches of the cell culture chamber, respectively, and the covered region of each kind of cell line was a separate part of the same one cell culture chamber (Figure 3c). The Figure 3a,b. showed the stained HRGECs under the 492/517 waves (excitation/emission) and the stained HepG2 cells under 350/461 waves (excitation/emission). Figure 3c. was the merged image with two kinds of stained cell lines. In addition, the results of cell count for the two kinds of cell lines indicated that the cell numbers of HepG2 cells and HRGECs were 457 ± 141 and 467 ± 101 in the same one cell culture chamber, respectively. The measurement of the growth region of the two kinds of cell lines showed similar results, where the growth area of HepG2 cells and HRGECs were (11.5 ± 2.1)% and (10.5 ± 3.5)%, respectively. The above results further demonstrated that the optimal dilution conditions of Section 3.1 were appropriate.

### 3.3. Growth Status of Bionic System

**Figure 4:**
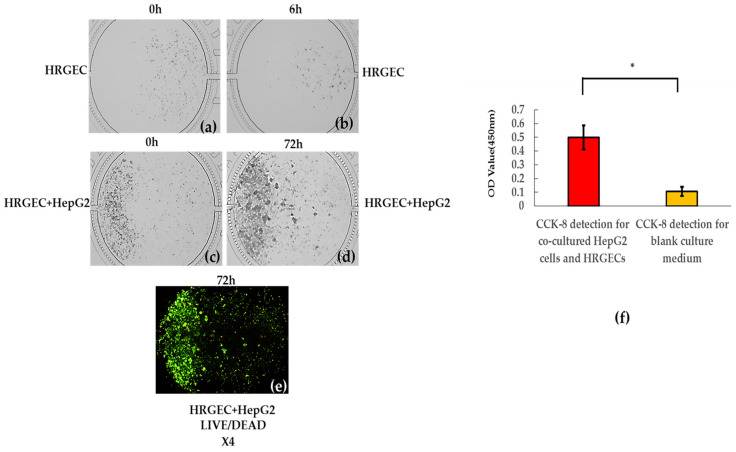
The observation of co-cultured cells and the CCK-8 detection of them. The images include: (**a**) at the 0th h after HRGEC cells covered the lower reaches of the cell culture chamber, (**b**) at the sixth h after HRGEC cells were cultured, (**c**) at the 0th h (in the first day) after HepG2 cells covered the upper reaches of the cell culture chamber, the numbers of HepG2 and HRGECs were 1072 ± 84 and 522 ± 250, respectively. (**d**) at the 72nd h (in the 3rd day) after the bionic system was cultured, the numbers of HepG2 and HRGECs were 2051 ± 971 and 703 ± 145, respectively, (**e**) the stained results of live/dead co-cultured cells, (**f**) the results of CCK-8 detection, *: compared with blank culture medium, *p* < 0.05.

Figure 4 shows that the HepG2 cells and HRGECs under dynamic co-culture could normally grow in a bionic system based on the microfluidic chip technique. At the first stage, the HRGECs realized adherent growth at the sixth h after they covered the lower reaches of the cell culture chamber (Figure 4b). The bionic system then continuously and dynamically grew within the subsequent 72 h (3 days) (Figure 4d). The cell counting results indicated that the numbers of HepG2 cells were respectively 1072 ± 84 and 2051 ± 971 on the first d and the third d, and that of HRGECs were respectively 522 ± 250 and 703 ± 145 within the same three days, which meant the bionic system appeared a proliferation trend within 3 days. After that, the CCK-8 results indicated that the OD value of the bionic system was significantly higher than that of the blank culture medium at the 72nd h (the 3rd d) after the bionic system was continuously and dynamically cultured (*t* = 7.398, *p* < 0.05) (Figure 4f). The coefficient of variation (CV) of CCK-8 detection was 7.36%, which verified that the improved and optimized bionic system was a stable and repeatable system in the microfluidic chip. After that, the stained results of live/dead co-cultured cells also displayed that the survival status of the bionic system were normal (Figure 4e). The above results demonstrated that the bionic system successfully realized a stable and normal culture, growth and proliferation in the microfluidic chip.

### 3.4. Influence of Emodin on Cell Viability of Bionic System

**Figure 5:**
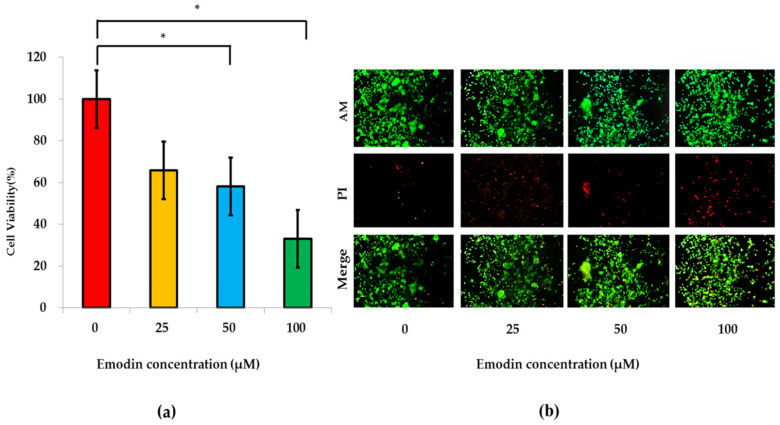
Influences of emodin exposure on the cell viability of a bionic system. (**a**) The cell viabilities of the bionic system gradually decreased with the increasing of the concentration of emodin exposure (from 0 to 100 μM) with the results of CCK-8 detection (n = 3). *: compared with 0 μM emodin group, *p* < 0.05. (**b**) The stained results of the live/dead co-cultured cells gradually changed with the increasing of the concentration of emodin exposure (from 0 to 100 μM). Live cells are in green and dead cells are in red.

The results of CCK-8 detection indicated that emodin exposure could gradually reduce the cell viability of a bionic system, and the cell viability of a bionic system under emodin exposures with different concentrations were 100%, 65.81%, 58.21% and 33.11%, respectively. The results of the further ANOVA analysis indicated that the differences of cell viability between 0 μM and 50 μM emodin exposure, and between 0 μM and 100 μM emodin exposure were significant (*p* < 0.05) (Figure 5a). On the other hand, the stained results of live/dead co-cultured cells also displayed that the amount of dead cells (red) gradually increased with the increasing of the concentrations of emodin exposure, and the amounts of dead cells at 50 μM and 100 μM emodin exposure were obviously larger than that at 0 μM emodin exposure (Figure 5b).

### 3.5. Influences of Emodin on the Basic Functions of Bionic System

**Figure 6:**
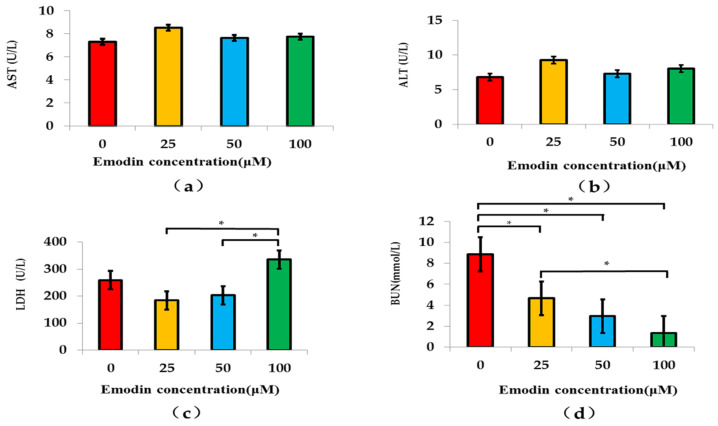
Influences of emodin exposure on the basic functions of bionic system. (**a**) AST activity, (**b**)ALT activity, (**c**) LDH activity, (**d**) BUN content; * compared with 0 μM (or 25 μM) emodin exposure, *p* < 0.05.

The results indicated that emodin exposure could not significantly affect the levels of AST and ALT of the bionic system (Figure 6a,b). The results of LDH detection showed a trend of increase after decrease with the increasing of the concentrations of emodin exposure, and the further ANOVA analysis indicated that the LDH values of 50 μM and 100 μM emodin exposure were significantly higher than that of 25 μM emodin exposure (*p* < 0.05) (Figure 6c). In addition, the BUN values of the bionic system significantly declined with increasing concentrations of emodin exposure, and the results of ANOVA analysis indicated that BUN values of 25 μM, 50 μM and 100 μM emodin exposure were significantly lower than that of 0 μM emodin exposure (*p* < 0.05). At the same time, the BUN value of 100 μM emodin exposure was also significantly lower than that of 25 μM emodin exposure (*p* < 0.05) (Figure 6d).

## 4. Discussion

The dynamic co-culture of multiple kinds of cell lines is a trend of a bionic system, which is expected to eventually replace conventional human experiments, animal experiments, and static culture with multiple kinds of cells. This technique has been used in some relative fields since 2016 [1,20,21,22,23], which involved the drug metabolism, the efficiency of the drug, and the development and evaluation of the drug. The results of the above mentioned studies point out that the dynamic co-culture with multiple kinds of cells can better simulate the microenvironment of human tissue, and reflect the interaction and metabolic process between different kinds of cell lines. Regrettably, this technique has not been used in the nutrition field until now. Our research is expected to make up for this deficiency and to conduct a beneficial exploration in evaluating the function and effect of ingredients in foods, which included but is not limited to the material of health food, through the optimization of the bionic system.

The bionic system of this research was composed of hepatocytes and renal cells, since the liver and kidney are the main organs that are involved in metabolism in evaluating health food [9]. Therefore, this bionic system of dynamic co-culture with HepG2 cells and HRGECs could form a relatively independent and integrated evaluation system. The HepG2 cell line is one kind of immortalized cell which is often used to conduct functional evaluation for different ingredients [24]. The HRGECs are an important part of the kidney, which mainly maintains the normal structure and function of the kidney [25]. Thus, the bionic system could preliminarily simulate the metabolic process of emodin from liver to kidney.

The routine operation of microfluidic cell chip culture analysis platform CellASIC ONIX2 could not achieve the effect of a bionic system of dynamic co-culture with HepG2 cells and HRGECs that simulated the metabolic process because the pressure of the sixth column could not realize the accurately separate and respective distribution of two kinds of cell lines in the same one cell culture chamber. According to our improved design, the HRGEC and HepG2 cells evenly and successively covered the same one cell culture chamber. This result meant that this improved design would contribute to form a relatively independent and complete bionic system that includes two kinds of different cell lines. In the bionic system, each kind of cell line can further contain two or three types of cells that belong to this kind of cell line, and the two kinds of cell lines that respectively grow at the upper and lower reaches of the same one cell culture chamber would be able to eventually grow into two organoids within one chamber, which will be a more complex and true bionic system. For example, we might perfuse a hepatic cell line that included the hepatic epithelial cell, hepatic stellate cell and liver sinusoidal endothelial cell into the upper reaches of the cell culture chamber after they are mixed, and perfuse a renal cell line that included HRGECs and renal tubular epithelial cell into the lower reaches of that after they are mixed. Finally, the two kinds of cell lines would grow into two separate organoids within one chamber. By then, not only “the effect of conventional co-culture” can be realized, but also the “the effect of two organoids” would be realized at the same time. Depending on this improved design, our team has applied for a patent for innovation, and we hope that this patent could contribute to the application of the CellASIC ONIX2 microfluidic cell chip laboratory in more fields.

Compared with conventional static cultures of cytology, the 3D culture with glue more closely resembles the true structure of human tissue. In addition, the glue could contribute to control the flow velocity of the cell solution which was perfused into the cell culture chambers of a microfluidic chip. As we know, both rat tail collagen I and gelatin have biocompatibility. Rat tail collagen I is the commonest collagen, which can promote the adherence and proliferation of cultured cells, especially the 3D culture for cells [26]. Gelatin possesses favorable physical properties including appropriate dispersion stability and water holding capacity [27], which can support the growth and proliferation of cells after they were digested by trypsin [28]. In this bionic system of our research, the rat tail collagen I and gelatin were mixed to simulate the independent structure and microenvironment of hepatocyte tissue and renal cell tissue, and also contribute to form a stably separate and respective distribution of each kind of cell line in the same one cell culture chamber. In Section 2.6, although single rat tail collagen I could not form a stably separate and respective distribution of each kind of cell line in the same one cell culture chamber, it still was added into gelatin to form mixed glue for providing auxiliary support in achieving better adherence and proliferation of cells. The results of subsequent experiments indicated that the mixed glue could meet the requirements of our research.

The results of Section 3.2 and Section 3.3 indicated that the improved and optimized bionic system could normally grow and proliferate, and had preliminarily simulated the flow process of fluid in the human body.

In this research, we chose emodin as representative to explore the influence of the materials of health food on this bionic system based on a microfluidic chip. The results of Section 3.4 indicated that the cell viability of the bionic system at 50 μM emodin exposure was significantly lower than that at 0 μM emodin exposure, which was different to the result of the other research of our team. The significant dosage of emodin exposure was 100 μM on the hepatocyte model based on the microfluidic chip in a previous study [8]. The reason for the difference of the significant dosage of emodin exposure between the two studies might be that the toxic reaction of 50 μM emodin exposure was more obvious for renal cells, which meant that most of the apoptosis might be in HRGECs of this bionic system, and emodin might be more dangerous for the kidney at this concentration.

In cytological study, the basically metabolic indicators of evaluating the effect of intervention usually included two aspects, which were enzyme indicators and metabolites. This research chose AST, ALT, LDH and BUN as the evaluation indicators to conduct a comprehensive evaluation of the bionic system. Among them, AST, ALT and LDH were used as enzyme indicators to evaluate the function of hepatocytes [29,30,31], and LDH was also used as an enzyme indicator to evaluate the function of renal cells [32,33]. In addition, the BUN was not only the metabolite of hepatocytes but also was that of renal cells [34]. Therefore, the detected results of the above indicators could basically reflect the intervened effects of emodin exposure on the bionic system. The results of AST and ALT levels of the bionic system hinted that we should appropriately extend the intervened time of emodin exposure in subsequent studies, which would be a new area of focus of our team. The LDH level of the bionic system appeared as a rising trend after a decline. This result was consistent with some other studies, which deemed that emodin possessed bidirectional effects on hepatocytes and renal cells, and it would protect hepatocytes and renal cells at appropriate concentrations or times, while it would damage them at high concentrations or over long times [9,13,16,17]. The BUN results indicated the time point that different kind of cell line releases BUN might be different after emodin exposure. This result needs to be further verified in our subsequent research.

The improved and optimized bionic system of dynamic co-culture with HepG2 cells and HRGECs has preliminarily realized the simulation for the biological process of emodin exposure in the human body through the improved routine operation of adding cells in a microfluidic chip, and through continuously and dynamically perfusing the culture medium and emodin solution. These results provided references and data for similar studies in the future. In addition, the results of the above experiments also indicated that this bionic system is able to be applied to evaluate more food ingredients besides the raw materials of health food, and to evaluate more functions of food ingredients besides toxic evaluation.

It also has been found that the profoundly metabolic mechanisms about the bionic system should be the research direction of our team in the future. We hope that our subsequent studies would eventually replace human trials, animal experiments and conventionally cytological experiments with static one cell line mono-cultures.

## 5. Conclusions

This research improved and optimized a bionic system of dynamic co-culture with HepG2 cells and HRGECs based on a microfluidic chip, and realized the evaluation for emodin by using this bionic system. We believe the optimized bionic system would provide a useful platform of in vitro alternative research, and the research results would provide an important reference for the reader. This bionic system would also be a novel alternative method of animal experiment and human trial in more aspects of nutritional evaluation in the future.

## Figures and Tables

**Table 1:** The design of different dilution conditions for HepG2 cells and HRGECs.

Combination	HepG2 Cells		HRGECs	
	Rat Tail Collagen I (mg/mL)	Gelatin (%)	Rat Tail Collagen I (mg/mL)	Gelatin (%)
a	−	2.5	1.5	−
b	−	4.0	3.0	−
c	−	5.0	4.0	−
d	−	7.5	−	7.5
e	−	8.5	−	8.5
f	−	10.0	−	10.0
g	−	−	−	−
h	1.3	7.5	1.3	7.5

Note: − without using this kind of glue.

## Data Availability

The data supporting reported results can be obtained from the corresponding author.
